# Intestinal Tropism of a Betacoronavirus (*Merbecovirus*) in Nathusius’s Pipistrelle Bat (*Pipistrellus nathusii*), Its Natural Host

**DOI:** 10.1128/jvi.00099-23

**Published:** 2023-03-01

**Authors:** Vera C. Mols, Mart M. Lamers, Lonneke ME. Leijten, Tim I. Breugem, Marco WG. van de Bildt, Petra B. van den Doel, Peter HC. Lina, Marion PG. Koopmans, Bart L. Haagmans, Thijs Kuiken, Lineke Begeman

**Affiliations:** a Department of Viroscience, Erasmus University Medical Center, Rotterdam, The Netherlands; b Department of Terrestrial Zoology, Naturalis Biodiversity Center, Leiden, The Netherlands; c Pandemic and Disaster Preparedness Centre, Erasmus University Medical Center, Rotterdam, The Netherlands; Loyola University Chicago - Health Sciences Campus

**Keywords:** chiroptera, viral tropism, coronavirus, betacoronavirus, pathogenesis, intestines

## Abstract

The emergence of several bat coronavirus-related disease outbreaks in human and domestic animals has fueled surveillance of coronaviruses in bats worldwide. However, little is known about how these viruses interact with their natural hosts. We demonstrate a *Betacoronavirus* (subgenus *Merbecovirus*), PN-βCoV, in the intestine of its natural host, Nathusius’s Pipistrelle Bat (*Pipistrellus nathusii)*, by combining molecular and microscopy techniques. Eighty-eight P. nathusii bat carcasses were tested for PN-βCoV RNA by RT-qPCR, of which 25 bats (28%) tested positive. PN-βCoV RNA was more often detected in samples of the intestinal tract than in other sample types. In addition, viral RNA loads were higher in intestinal samples compared to other sample types, both on average and in each individual bat. In one bat, we demonstrated *Merbecovirus* antigen and PN-βCoV RNA expression in intestinal epithelium and the underlying connective tissue using immunohistochemistry and *in situ* hybridization, respectively. These results indicate that PN-βCoV has a tropism for the intestinal epithelium of its natural host, Nathusius’s Pipistrelle Bat, and imply that the fecal-oral route is a possible route of transmission.

**IMPORTANCE** Virtually all mammal species circulate coronaviruses. Most of these viruses will infect one host species; however, coronaviruses are known to include species that can infect multiple hosts, for example the well-known virus that caused a pandemic, SARS-CoV-2. Chiroptera (bats) include over 1,400 different species, which are expected to harbor a great variety of coronaviruses. However, we know very little about how any of these coronaviruses interact with their bat hosts; for example, we do not know their modes of transmissions, or which cells they infect. Thus, we have a limited understanding of coronavirus infections in this important host group. The significance of our study is that we learned that a bat coronavirus that occurs in a common bat species in Europe has a tropism for the intestines. This implies the fecal-oral route is a likely transmission route.

## INTRODUCTION

Several coronaviruses (CoVs), including severe acute respiratory syndrome CoV type 1 (SARS-CoV-1), Middle East respiratory syndrome CoV (MERS-CoV), severe acute respiratory syndrome CoV type 2 (SARS-CoV-2), and swine acute diarrhea syndrome CoV (SADS-CoV) have caused notable disease outbreaks in humans and animals over the last 20 years (1, 2). Phylogenetic analyses suggest that these viruses originated, at least partly, from bat CoVs (3–6), which likely spilled over from bats to humans and pigs, either directly or through an intermediate host (3, 7, 8). Bats are the second largest order of mammals, comprising approximately 20% of all mammalian species (9, 10). The number of zoonotic viruses originating from this large mammalian order is proportional to the number of different species it is comprised of (11). When bats are sampled and tested, coronaviruses are frequently detected, suggesting bats are suitable hosts for coronaviruses (7, 12–21). Bat coronaviruses that have been detected belong to the *Alphacoronavirus* and *Betacoronavirus* genera, two of the four genera within the *Coronaviridae* family. Phylogenetics of bat coronaviruses seem to follow the phylogenetics of bat species, suggesting virus-host coevolution (22). Currently there is little known about what characteristics make bat coronaviruses more likely to cross the species barrier and transmit outside their normal bat species host.

The tropism of bat coronaviruses is largely unknown. Tropism for the intestinal tract has been suggested based on the high rate of RNA detection in fecal samples and intestinal tissues (7, 12, 23–26). However, detection of viral RNA in feces does not necessarily prove that the intestinal tract is the primary replication site. For example, RNA of human influenza virus and bat lyssavirus type 1 also can be detected in feces, even though their primary replication sites are the respiratory tract and nervous system, respectively (27, 28). Bat coronavirus RNA has also been detected in upper and lower respiratory tract samples of bats (29, 30). A bat coronavirus found in a naturally infected, captive Little Brown Bat (*Myotis lucifugus*) colony shows tropism for the respiratory tract, more specifically in bronchial epithelial cells (31). In humans, for which coronavirus infections are relatively well studied, coronaviruses can show a tropism for both the upper and lower respiratory tract as well as the intestinal tract (32, 33). In mice, mouse hepatitis virus has a tropism for the liver, nervous tissue, intestinal tract, and lower respiratory tract, depending on the virus strain (34, 35). In livestock and companion animals, coronaviruses commonly cause infections in the respiratory or digestive tract, or both (32, 36–38). Of these, feline enteric CoV, as well as ferret enteric CoV, which replicate predominantly in the intestinal tract, specifically in the intestinal epithelium, can mutate into variants with tropism for the immune system, specifically in macrophages (39, 40), causing systemic infections (41). Given the diversity of CoV tropism in above species, a similar diversity may be expected for the tropism of bat CoVs in their chiropteran hosts.

Bats have been identified as ancestral hosts for epidemic, newly emerging human, and domestic animal coronaviruses. However, determinants of spillover probability and pathways from bat CoVs to other species are largely unknown. Viral dynamics, such as tissue tropism within a reservoir host, are important contributing factors to external shedding and consequently mode of exposure to susceptible animals (42). To better understand the tissue tropism of coronaviruses in bats, our study aimed to investigate the tissue tropism of a β-CoV regularly detected in Nathusius’s Pipistrelle Bats *(Pipistrellus nathusii)* (L. Begeman), a bat species commonly found in the Netherlands (43). This virus, PN-βCoV (subgenus *Merbecoviruses*, previously known as lineage 2c), has been previously detected by others in Pipistrelle Bats (44, 45).

## RESULTS

### Detection and quantification of PN-βCoV RNA in bats.

Overall, 28% (25 of 88) of bats tested positive for PN-βCoV RNA. RNA was detected in intestinal tissues of 21 of 83 (25%) bats for which it was available, in rectal swabs of 16 of 88 (18%) bats, in nose washes of 3 of 37 (8%) bats for which it was available, and in the lungs of 3 of 79 (3%) bats for which it was available (Supplement S1). The percentage of bats testing positive was significantly higher for bats that were necropsied directly after death (8 of 11 bats, 73% positive), compared to bats necropsied after a period of storage at −20°C (17 of 73 bats, 23% positive, χ^2^, *P* < 0.001, Table 1).

**TABLE 1:** Comparison of detection rates and RNA loads detected by RT-qPCR targeting a 154 bp fragment of *Merbecovirus* UpE gene in intestinal tissue, stored dry or in RNAlater (RL), and in rectal swabs, lung tissues and nose wash specimens of bat carcasses necropsied shortly after death versus bat carcasses frozen prior to necropsy

	Intestine (dry)		Intestine (RL)		Rectal swab		Lung		Nose wash	
Group	Percentage positive (no. positive/total)	Mean *Ct* ± SD	Percentage positive (no. positive/total)	Mean *Ct* ± SD	Percentage positive (no. positive/total)	Mean *Ct* ± SD	Percentage positive (no. positive/total)	Mean *Ct* ± SD	Percentage positive (no. positive/total)	Mean *Ct* ± SD
Frozen (*n* = 74)	18% (9/49)	28 ± 4.45	26% (12/46)	30 ± 3.83	14% (10/74)	28 ± 3.68	4% (3/68)	33 ± 2.63	7% (2/27)	34 ± 2.60
Fresh (*n* = 14)	60% (6/10)	27 ± 5.34	50% (3/6)	23 ± 5.50	43% (6/14)^*a*^	28 ± 3.94	0% (6/11)	NA^*b*^	10% (1/10)	32 ± 0
Combined (*n* = 88)	25% (15/59)	28 ± 4.65	29% (15/52)	28 ± 4.75	18% (16/88)^*a*^	28 ± 3.65	4% (3/79)	33 ± 2.63	8% (3/37)	33 ± 2.21

a No intestinal tissue was available for virological analysis in 5 bats, one of which tested positive in rectal swab. The other four were excluded from further analysis.

b NA, not applicable.

The majority of bats (15 of 25, 60%) tested PN-βCoV RNA positive in exclusively intestinal samples. We did not detect bats that were positive in only the respiratory tract samples. In addition, intestinal samples tested positive more frequently compared to any other sample origin (57 to 79% compared to 4 to 20% respiratory sample types and 12 to 16% other sample types, Table 2). Furthermore, higher levels of viral RNA (mean *Ct* values of 25 to 28) were detected in intestinal sample types compared to all other sample types (mean *Ct* values of 30 to 34). Still, all other, nonintestinal sample types included in our analyses had one or more positives, with *Ct* values ranging from 23 (kidney) to 37 (also kidney) (Table 2).

**TABLE 2:** Proportion *Merbecovirus* positives per sample type in *Merbecovirus*-positive bats, and RNA loads detected by RT-qPCR. Totals indicate the total number of bats positive in at least one sample within a group of sample types

Sample type	Percentage positive (no. positive/total)	Mean *Ct* value (range)
Intestinal		
RNAlater intestine	79% (15/19)	28 (18–36)
Dry intestine	63% (15/24)	28 (20–37)
Rectal swab	64% (16/25)	28 (23–33)
Feces	57% (12/21)	25 (17–35)
**Total**	100% (25/25)	
Respiratory		
Nose wash	20% (3/15)	33 (32–36)
Lung	12% (3/25)	33 (31–36)
Oral swab	4% (1/25)	31 (31)
**Total**	16% (4/25)	
Other		
Liver	16% (4/25)	30 (24–33)
Kidney	16% (4/25)	32 (23–37)
Brain	16% (4/25)	34 (32–35)
Spleen	12% (3/25)	33 (28–36)

The following bivariate statistical analyses to test for a relation between a positive *Merbecovirus* outcome and sex, do not include four bats from which no intestinal tissue was available. Bats included in this study consisted of 66 (79%) males and 18 (21%) females. Detection of PN-βCoV RNA was not significantly different between males (30%, 20 of 66 positive) and females (28%, 5 of 18 positive) (χ^2^, *P* = 0.835). Because our results showed a significantly higher percentage of bats testing positive when in bats necropsied shortly after death compared to bats necropsied after storage at −20°C, sex bias was also tested in the two groups separately. Again, no significant differences were detected between males and females in either of those groups (frozen stored: males 25%, 14 of 57 positive; females 19%, 3 of 16 positive; χ^2^, *P* = 0.627; direct necropsy: males 67%, 6 of 9 positive; females 100%, 2 of 2 positive; χ^2^, *P* = 0.338).

### Phylogenetic analysis.

**FIG 1:**
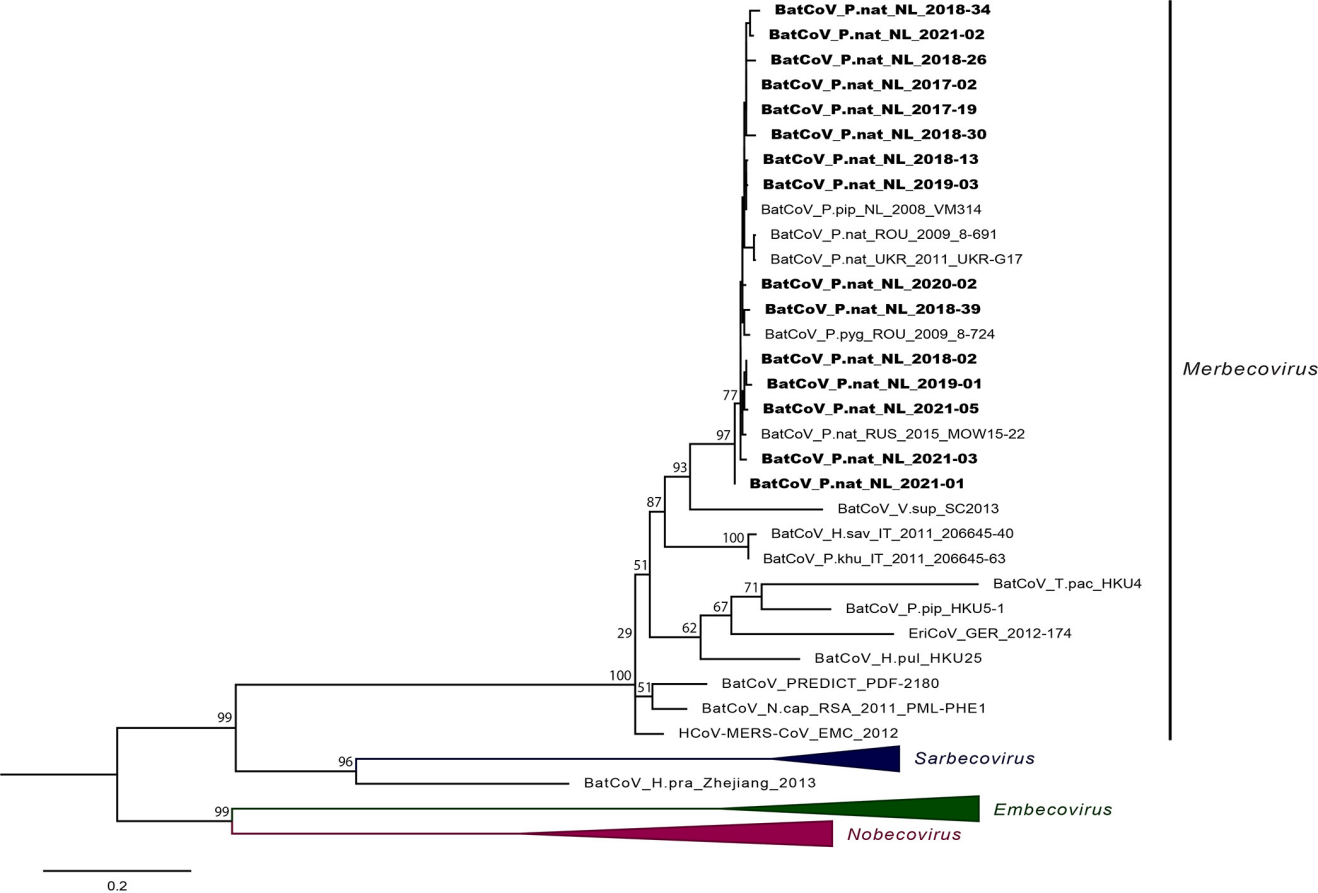
RNA-dependent RNA polymerase-based phylogeny of novel sequences and reference *Betacoronaviruses* obtained from GenBank based on 338–396 nt fragments corresponding to positions 15353 to 15747 in Middle East respiratory syndrome-related coronavirus isolate Bat-CoV/P.nathusii/Russia/MOW15-22/2015 (GenBank accession no. ON325306). Phylogenetically distinct PN-βCoV sequences obtained in this study are displayed in bold. Reference sequences of *Betacoronaviruses* classified within subgenera Sarbecovirus, Embecovirus and Nobecovirus are collapsed. Accession numbers available in Supplement S2 and S3.

*Rdrp* fragments of 395 to 396 nt in length were obtained from samples of 13 RT-qPCR-positive bats, as well as a 359 and 383 nt *Rdrp* fragment from two other RT-qPCR-positive bats (Supplement S2). Obtained 395 to 396 nt of *Rdrp* fragments showed a sequence similarity of 96.7 to 100% among one another. Phylogenetic analysis showed that PN-βCoV clustered with other previously detected *Merbecoviruses* (Fig. 1) in *Pipistrellus* species found in The Netherlands, Romania, Ukraine, and Russia (Supplement S3), with a sequence similarity of ≥97%.

### Immunohistochemistry and *in situ* hybridization.

**FIG 2:**
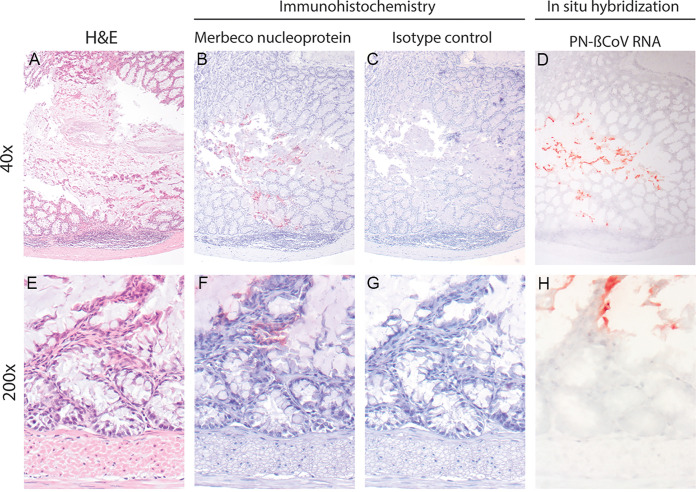
Histology, PN-βCoV viral antigen expression and PN-βCoV viral RNA expression at two magnifications, in the intestinal mucosa of a Nathusius’s Pipistrelle Bat. (A) Intestine with lumen in the center and epithelium on top and bottom, lined by lamina propria, muscular wall and serosa on the bottom. The lamina propria is expanded by densely cellular gastrointestinal lymphoid tissue. Epithelium nearest to the lumen is sloughed off due to autolysis, while more toward the serosa epithelial architecture has been preserved. H&E. (B) PN-βCoV antigen expression, visible as red staining, in a distinct intestinal epithelial area that extends from the center (lumen side) to the bottom of the panel (serosa side). IHC with MERS-CoV nucleoprotein antibody which shows cross-reactivity with PN-βCoV, developed with AEC. (C) Isotype control shows no staining. Serial section, isotype control with anti-mouse IgG1. (D) Expression of PN-βCoV RNA is present as red staining in the same area staining positive for PN-βCoV antigen. Serial section, ISH for PN-βCoV RNA. (E) Higher magnification of intestinal mucosa at another location than shown in (A) to (D), with epithelial layer containing crypts embedded in connective tissue (lamina propria) on top and muscular layer on the bottom of the panel. No pathological changes, such as infiltration of immune cells or necrosis. Mild autolysis at the base of the crypts, progressing to moderate autolysis toward the top of the panel. H&E. (F) PN-βCoV antigen is present as dark red granules in cells within intestinal lamina propria and throughout the cytoplasm of epithelial cells. Serial section, IHC with MERS-CoV nucleoprotein antibody which shows cross-reactivity with PN-βCoV, developed with AEC. (G) Isotype control shows no staining. Serial section, isotype control with anti-mouse IgG1. (H) Expression of PN-βCoV RNA is present as red staining in the same area that expresses PN-βCoV antigen. The histological details are faint due to the harsh treatment of the tissue section for ISH. Serial section, ISH for PN-βCoV RNA.

Of 23 viral RNA positive bats with sufficiently fresh tissue to allow histological analysis, 1 bat expressed viral antigen in cells within the intestine (Fig. 2). In all other tissues of this bat, and all tissues of the other 22 bats, viral antigen was not detected. Tissues from 2 of 25 PN-βCoV RNA-positive bats showed advanced autolysis of the epithelium and were excluded from IHC staining.

**FIG 3:**
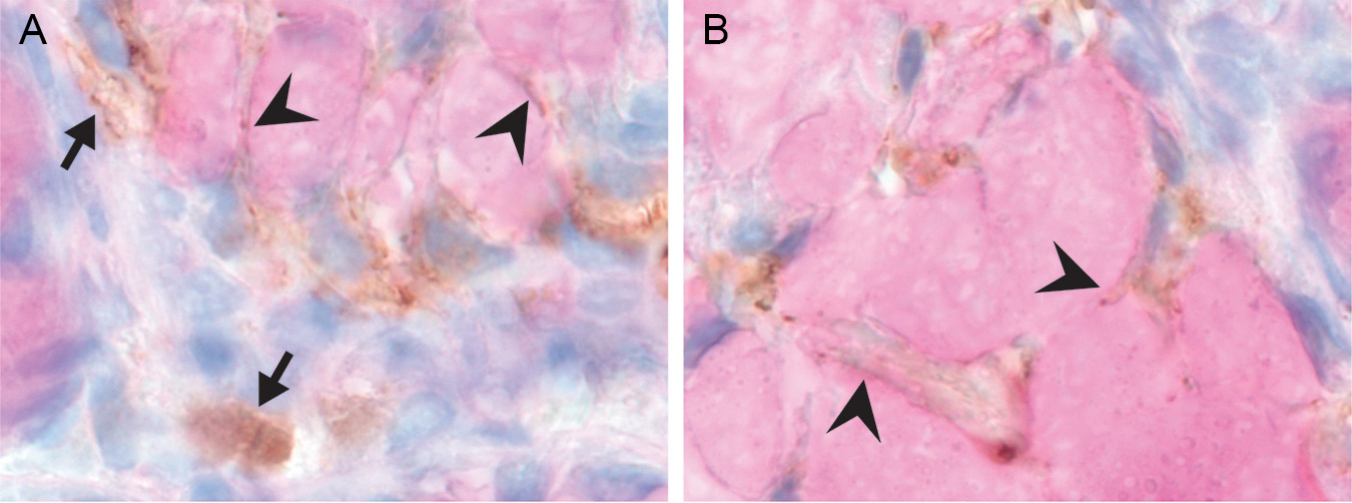
PN-βCoV antigen expression in the intestine. Arrowheads indicate brown virus-antigen staining that based on localization and morphology is consistent to be in cytoplasm of mucus-cells. Mucus-cells are characterized by a basal, somewhat flattened nucleus, and a thin layer of cytoplasm that surrounds a purple globule (mucus). Arrows indicate brown virus staining of cells in the connective tissue bordering crypts (lamina propria), potentially fibroblasts, or immune cells (×1000 magnification, PN-βCoV IHC developed with DAB, with PAS counterstain to highlight mucus inside mucus-cells).

In the one viral antigen positive bat, antigen was expressed as variably sized, indistinctly delineated granules within cytoplasm of both individual cells and clusters of cells, which displayed a morphology consistent with mucus producing cells. In addition, scattered, individual cells in the connective tissue (lamina propria) between the intestinal crypts (Fig. 2 and 3) stained similarly positive. The cells expressing viral antigen within the lamina propria were difficult to identify based on morphology and could either be fibroblasts or immune cells. The isotype control to test for aspecific binding was negative. There was no inflammation or other lesions that colocalized with the viral antigen expression.

*In situ* hybridization on serial sections of this intestinal sample expressing viral antigen showed PN-βCoV RNA expression in the same region where there was viral antigen expression. Viral RNA was visible as bright pink granules in the same area that expressed viral antigen (Fig. 2). Comparison of the viral RT-qPCR results of this bat with those of the viral antigen negative bats showed that viral loads were higher, especially in the intestinal samples: *Ct* 18 in intestine stored in RNAlater, compared to average *Ct* of 28 (range 18 to 36); *Ct* 19 in feces compared to 25 (range 17 to 35); *Ct* 23 in dry intestine compared to 28 (range 20 to 37); and *Ct* 26 in rectal swab compared to 28 (range 23 to 33). The other (IHC negative) tissues of this bat had *Ct* values of 23 (kidney), 24 (liver), and 28 (spleen). Supplement S1 provides RT-qPCR results for each individual positive bat.

## DISCUSSION

By the demonstration of expression of viral antigen and PN-βCoV RNA in epithelial cells and cells within the lamina propria of the intestine of one bat, we show PN-βCoV has intestinal tropism in its natural bat host, Nathusius’s Pipistrelle Bat. Our results suggest these cells are a replication site for this virus and a likely source for the viral RNA detected in feces and rectal swabs of other bats in this study. The detection of higher viral RNA loads in intestinal samples compared to any other sample type in all positive bats support this. Based on morphology virus positive cells in the intestinal epithelium included mucus-producing cells, and those in the lamina propria were either fibroblasts or immune cells. Further characterization of these cell types would increase our understanding of this virus infection in Nathusius’s Pipistrelle Bats.

While we detected intestinal tract tropism for PN-βCoV in our Nathusius’s Pipistrelle Bats, others have detected a bat coronavirus that had respiratory tract tropism in its natural bat hosts. In that study ‘Myl-CoV’ RNA, was somewhat similar to our results detected in intestinal tissues of a high percentage (53 of 174, 30%) of Little Brown Bats, and for a much smaller percentage in lungs (5 of 174, 3%). In that study, however, only lung sections expressed coronavirus antigen by IHC, and not sections of the intestinal tract. In the lungs, bronchial epithelial cells specifically stained positive for virus antigen, and the presence of virus particles in these cells was confirmed by electron microscopy (31). Coronavirus infections in other mammals, like humans and cattle, have shown that coronaviruses can have a tropism for the respiratory tract as well as the intestinal tract in a single host species (33). Future studies of coronaviral tropism in infected bats will help to understand if the PN-βCoV in Nathusius’s Pipistrelle Bats has additional tropism for cells outside the gastrointestinal tract.

Coronavirus tropism is highly dependent on the presence and distribution of cellular receptors (46–48). The cellular receptors used by PN-βCoV are currently unknown. However, *in silico* and phylogenetic analysis suggests that this virus might have affinity for bat DPP4 (49). DPP4 expression has been demonstrated in epithelial cells of the intestinal tract, but not in the respiratory tract, in a species closely related to Nathusius’s Pipistrelle Bats (50). This suggests that coronaviruses that use DPP4 will have an intestinal tropism in these bats.

Our findings indicate intestinal tropism; however, several other intestine samples tested positive by RT-qPCR yet showed no viral antigen expression by IHC. A possible explanation is that the IHC is not very sensitive. However, the overlap in staining area of the PN-βCoV ISH and virus IHC, together with the known high sensitivity of *in situ* hybridization technique (51), contradicts this. A possible explanation for these results is that our virus IHC was less sensitive than our viral RT-qPCR, so that only cells at their peak of virus production could be detected. Alternatively, the amount of intestine in the slides might not have been big enough to detect a small number of infected cells with IHC. Another explanation is that viral RNA can persist long after viral infection has been cleared. Similarly, it has been observed that SARS-CoV-2 infected patients can remain PCR positive up to months after disease recovery. Many of these PCR positive patients that do not show clinical signs of COVID19 are culture negative and do not transmit SARS-CoV-2; nor does SARS-CoV-2 infection reemerge later (52–54), suggesting that they cleared their infection, while viral RNA remains detectable in their samples for some time. For SARS-CoV-2 infection and likely other virus infections, another determinant of virus infection, besides detection of antigen positive cells, is the detection of high viral loads by quantitative PCR (55). In our study the intestine sample in which we detected virus antigen had the highest viral load measured by quantitative PCR. This suggests that, for future studies into bat coronavirus infection, quantitative viral PCRs are a helpful tool to add to the currently commonly performed qualitative viral PCRs.

To conclude, PN-βCoV was detected in epithelial cells of the intestine in its natural host, Nathusius’s Pipistrelle Bat, suggesting this is a replication site for the virus. The fecal-oral route therefore represents a likely transmission route among bats of this species. Revealing intestines as a site of virus replication will help to focus future efforts to culture PN-βCoV, to understand its pathogenesis, and to understand transmission of the virus between bats.

## MATERIALS AND METHODS

### Study setup.

The study was embedded in a bat mortality screening program further described below. To investigate the tropism of a coronavirus-bat host pair, we chose a β-CoV that we detected in naturally infected Nathusius’s Pipistrelle Bats. We chose a β-CoV because previously recognized promiscuous, less species-specific coronaviruses like SARS-CoVs, MERS-CoV, and bovine coronavirus belong to this genus (56), and we chose Nathusius’s Pipistrelle Bats because carcasses of naturally infected bats were available to us, and because it is one of the most common synanthropic bat species in the Netherlands, mainly from mid-August until mid-May. From mid-May until mid-August, the majority of the population consist of males, and females are almost absent. In late summer and early autumn, there is an influx of large numbers of adult and subadult females into the Netherlands to join territorial males to mate (43). First, we identified carcasses for the presence of P. nathusii β-CoV (PN-βCoV) RNA by investigating intestinal and respiratory samples using a RT-qPCR set up for the detection of this *Merbecovirus*. Intestinal samples have proven to be a sensitive sample to detect viruses with varied tropism, including respiratory (28), neurologic (27), and intestinal tropism (57). Next, a standard set of samples from each positive carcass was further investigated by RT-qPCR and immunohistochemistry to localize PN-βCoV RNA and antigen. This standard set contained all tissues that were available for virology and microscopy in parallel, which were lung, intestine, kidney, brain, liver, and spleen. PN-βCoV specific *in situ* hybridization was used to confirm IHC-staining specificity. If intracellular virus antigen or viral RNA was detected by staining, colocalization with lesions was examined on serial sections stained with hematoxylin and eosin (H&E).

### Bat sampling and necropsy.

We used carcasses from Nathusius’s Pipistrelle Bats that were found dead, or from bats that were euthanized because of poor prognosis for survival and release, based on evaluation by bat rehabilitators in rehabilitation centers across the Netherlands. These bat carcasses were routinely frozen at −20°C until the date of transport to our facility and subsequent necropsy. Transport and investigation were performed under permit FF/75A/2015/036 from the Dutch Ministry of Economic Affairs. In response to the initiation of this project, a request was made to a local rehabilitation center to also immediately notify the researchers if a Nathusius’s Pipistrelle Bat in their care had died. These bats were then necropsied as soon as possible, in order to obtain non-frozen tissues in addition to the tissues of the frozen carcasses. For this study, all 88 Nathusius’s Pipistrelle Bat carcasses collected between 2016 and 2021 were selected. Of these, 74 had been stored at −20°C between 1 and 23 months prior to necropsy, and 14 had been necropsied within 6 h postmortem. Species of each bat was determined by an experienced bat researcher (PHCL) using phenotypic characteristics, and later confirmed by DNA analysis.

Necropsies were performed according to standard protocol at biosafety level 2. Samples collected for virology and microscopy in parallel were lung, intestine, kidney, brain, liver, and spleen. Additionally, rectal and pharyngeal swab specimens and, if present, feces from the rectum, were collected for virological analysis and were each stored in 1 mL virus transport medium (VTM) made according to an existing protocol (58). Nasal washes were performed for a subset of carcasses, arbitrarily selected. For this, 500 μL VTM was gently inserted into one of the choana, and then collected when dripping from the nostrils. For virology, samples were stored at −80°C, or in RNAlater (4°C for 24 h to fixate, −20°C storage). For microscopy, samples were fixed in formalin and paraffin embedded. These included small pieces of lung, brain, kidney, liver and spleen. Complete intestines were embedded, except for a centimeter distal segment that was collected for virology. Skulls were fixed in formalin, then decalcified in 10% EDTA before dehydration and embedding in paraffin in the sagittal plane in order to get a good view on the entire nose.

### Molecular confirmation of bat species.

For confirmation of the bat species via DNA analysis, liver tissue from each bat was homogenized in 1 mL VTM using a 1/4” ceramic sphere (MP Biomedicals). DNA was extracted from 200 μL homogenate using a QIAamp DNA minikit (Qiagen) in accordance with the manufacturer’s instructions and eluted in 200 μL buffer AE provided in the kit. 5 μL eluted DNA was added to a PCR mix containing 0.75 μL (each) of 10 μM primers (Fwd primer SFF_145: 5′ GTHACHGCYCAYGCHTTYGTAATAAT 3′, Rv primer SFF_351r: 5′ CTCCWGCRTGDGCWAGRTTTCC 3′) targeting a 202 nt fragment of mitochondrial gene cyto-chrome c oxidase subunit I (COI) (59), 0.75 μL 10 mM deoxynucleoside triphosphates, 2.5 μL 10× PfuUltra II Rxn buffer (Agilent), 0.5 μL PfuUltra II fusion HS DNA polymerase (Agilent), 0.5 μL dimethyl sulfoxide (Sigma-Aldrich), and 14.25 μL MilliQ water. Amplification was performed in a C1000 Touch Thermal Cycler (Bio-Rad) using the following program: 3 min at 95°C, 40 cycles of 30 s at 95°C, 30 s at 48°C and 40 s at 72°C, and a final extension step of 10 min at 72°C. 5 μL PCR product was reamplified using the same PCR mix and program.

The second-round PCR products were run on a 2% agarose gel with SYBR Safe DNA Gel Stain (Invitrogen) and visualized using a ChemiDoc MP imaging system (Bio-Rad). Bands with the expected size of ~202 nt were excised on a UVP Visi-Blue Transilluminator and stored at −20°C until further processing.

DNA was extracted from excised bands using a QIAquick Gel Extraction kit (Qiagen) in accordance with the manufacturer’s instructions and eluted in 10 μL Buffer EB (10 mM Tris·Cl, pH 8.5) provided in the kit. A sanger sequencing PCR was performed in two 10 μL reactions (one forward and one reverse per sample) containing 2 μL DNA extract, 3 μL primer (2 pmol/μL) (forward reaction primer SFF_145, reverse reaction primer SFF_145), 1.75 μL 5× sequencing buffer (Applied Biosystems), 0.5 μL BigDye Terminator v3.1 Ready Reaction Mix (Applied Biosystems) and 2.75 μL MilliQ water. Sanger sequencing PCR was performed in a C1000 Touch Thermal Cycler (Bio-Rad) using the following program: 10 s at 96°C, 30 s at 45°C and 30 cycles of 4 min at 60°C. Products were analyzed using an Applied Biosystems Hitachi 3130xl Genetic Analyzer (Applied Biosystems). Sequences generated were assembled and trimmed in DNA Baser Assembler v5.15.0 and compared to available sequences in GenBank.

### RT-qPCR.

Primers and a probe were designed using PN-βCoV (accession no. OQ405399 and OQ405401) and a MERS-CoV reference sequence (accession no. NC_019843.3) to amplify and quantify PN-βCoV Up-E gene as well as MERS-CoV Up-E gene via RT-qPCR. To evaluate specificity, *in silico* analysis was performed through alignment of various *Merbecovirus* reference sequences as well as UpE gene of an *Alphacoronavirus* detected in P. nathusii bats (accession no. OQ405400). Primers were able to bind exclusively to PN-βCoV and MERS-CoV Up-E genes. RT-qPCR was performed on rectal swabs and intestinal tissue from each bat, as well as lung and nose wash. Intestine in RNAlater was washed in PBS and homogenized in 1 mL VTM using a 1/4” ceramic sphere (MP Biomedicals). Intestine stored without medium was used if intestine in RNAlater was not available. Total nucleic acids were isolated from 60 μL rectal swab suspension and 60 μL intestine homogenate supernatant via a previously in-house developed method using magnetic beads (AMPure XP, Beckman Coulter) and MagNA Pure 96 External Lysis Buffer (Roche) (60) and eluted into 30 μL elution buffer (Roche). Three μL of a known concentration of PDV was added to the lysis buffer as internal control; 5 μL RNA extract was added to 15 μL RT-qPCR mix targeting a 154 bp fragment of virus UpE gene containing 0.5 μL of 10 μM concentrations of Fwd primer (5′-CCTGCAACGCGCGAT-3′), Rv primer (5′-TGGACAAAGGGTAACATAGTTCG-3′) and probe (5′ FAM-TGGATTAGCCTCTACACGGGACCCATAG-3′), 0.4 μL PDV primer/probe stock, 5 μL of TaqMan Fast Virus 1-Step Master Mix (ThermoFisher Scientific), and 8.1 μL MilliQ water. 5 μL eluted RNA from MERS-CoV culture supernatant derived from infected human airway organoids, diluted 1:10 in MilliQ water, was used as a positive control. Amplification was performed and detected using the following cycling program on an Applied Biosystems 7500 Real Time PCR System: 5 min at 50°C and 20 s at 95°C, followed by 40 cycles of 3 s at 950C and 31 s at 60°C. Of all bats in which virus RNA was detected (Ct <40) in intestine, rectal swab, lungs, or nose wash, all other available samples were further tested for PN-βCoV RNA.

### Coronavirus PCR and sequencing.

In order to obtain viral sequences of partial RNA-dependent RNA polymerase (*Rdrp*) for each RT-qPCR-positive bat, samples with the highest viral RNA load based on the RT-qPCR were further analyzed with a pan-coronavirus PCR as described by (61). Sanger sequencing was performed using a BigDye Terminator Cycle v3.1 kit in accordance with the manufacturer’s instructions in an Applied Biosystems Hitachi 3130xl Genetic Analyzer. Assembly was performed using DNA Baser Assembler v5.15.0. Sequences were aligned using ClustalW with reference sequences from GenBank, and a maximum-likelihood tree was obtained using IQ-TREE multicore v1.6.12 and visualized with FigTree v1.4.4.

### Statistical analysis.

Bivariate X^2^-tests (*P* < 0.05) were performed to exploratively evaluate possible influence of bat sex and carcass storage conditions, namely: on ice for up to 6 h prior to necropsy (fresh) or stored for a period of time at −20°C (frozen), on PN-βCoV RNA detection.

### Immunohistochemistry.

*Merbecovirus* immunohistochemistry was performed only on those PN-βCoV RNA positive bats for which tissues showed minimal autolytic changes (23 of 25) in hematoxylin and eosin (H&E) stained sections. For immunohistochemistry, we used serial, 3-μm-thick, formalin-fixed, paraffin-embedded sections of all available tissues. Lung tissue derived from a MERS-CoV infected mouse was used as a positive control. Sections were deparaffinized and pretreated by boiling in citric acid buffer (10 mM, pH 6.0) for 15 min. Sections were then washed twice with PBS and endogenous peroxidase was blocked with 3% hydrogen peroxide. After washing with PBS and PBS/Tween, the sections were stained with 5 μg/mL mouse-anti-HCoV-EMC/2012 nucleoprotein mIgG1 (Sino Biological Inc. Cat No. 44068-MM10) diluted in PBS/0.1% BSA overnight at 4°C. As a negative control, consecutive sections were stained with mouse IgG1 isotype control 1:100 in PBS/0.1% BSA. Slides were washed twice with PBS/Tween and incubated with anti-mIgG1-HRP diluted 1:100 in PBS/0.1% BSA for 1 h at room temperature, then washed twice with PBS. Staining was developed with 5% aminoethyl carbazole (AEC) in DMF and 0.05% H_2_O_2_ in NaAc. Sections were counterstained with Mayer’s hematoxylin.

To better visualize morphology of cells in which viral antigen had been detected, the *Merbecovirus* immunohistochemistry protocol described above was repeated on sections of the *Merbecovirus* antigen-positive bat intestine, but with a Periodic acid-Schiff (PAS) counterstain instead of Mayer's hematoxylin. To ensure contrast with the purple-magenta PAS counterstain, the IHC was developed with 3,3′-diaminobenzidine (DAB), which stains brown, instead of the previously used AEC, which stains red. Micrographs were taken with a ZEISS Axiocam 305 color camera attached to a ZEISS Axio Imager.A2 light microscope.

### *In situ* hybridization.

To confirm specificity of the IHC staining, IHC-positive bat tissues were selected for *in situ* hybridization. ISH was performed on 5 μm FFPE sections according to manufacturer’s instructions for BaseScope. Lung tissue derived from a mouse inoculated with MERS-CoV was used as positive control. A C1 BaseScope (Advanced Cell Diagnostics) probe was custom designed to target a conserved region of PN-βCoV orf1ab, in addition to MERS orf1ab. Consecutive slides for each section were stained with a DAPB probe (Advanced Cell Diagnostics Cat No. 701011) as negative control. Micrographs were taken using a ZEISS Axiocam 305 color camera attached to a ZEISS Axio Imager.A2 light microscope.

### Data availability.

The PN-βCoV (partial) genome sequences reported in this study were deposited to GenBank under accession numbers OQ405399 and OQ405401. The *Alphacoronavirus* genome was deposited under accession number OQ405400. The *Rdrp* sequences were deposited under accession numbers OQ348392 to OQ348406.
