# Supplementary material for: Intestinal Tropism of a Betacoronavirus (Merbecovirus) in Nathusius’s Pipistrelle Bat (Pipistrellus nathusii), Its Natural Host
Source: J Virol. 2023 Mar 1;97(3):e00099-23. doi: 10.1128/jvi.00099-23 (PMC10062147; doi:10.1128/jvi.00099-23)
Supplement: Supplemental file 1 — Supplemental material. Download jvi.00099-23-s0001.pdf, PDF file, 1.3 MB [file jvi.00099-23-s0001.pdf]

S1: List of tested *Pipistrellus nathusii* bats with biological variables and PN-ßCoV RT-qPCR results. N/A = not available. NT = not tested.

			Date dead			Date found alive										Ct values										
Erasmus nr.	PN-ßCoV Positive	Found alive	Day	Month	Year	Day	Month	Year	Season found	Euthanazised	Frozen prior to necropsy	Weight (g)	Forearm length (mm)	Fat reserves	Sex	Intestine	Intestine in RNAlater	Feces	Rectal swab	Lung	Liver	Kidney	Spleen	Brain	Oral swab	Nose Wash
PN180308.03	No	Yes	24	1	2017	24	1	2017	Winter	N/A	Yes	5	NT	Mild	M	UD	N/A	N/A	UD	UD	NT	NT	NT	NT	NT	N/A
PN180308.17	Yes	Yes	28	1	2017	27	1	2017	Winter	No	Yes	4	NT	Moderate	M	**27**	N/A	**26**	**30**	UD	UD	UD	UD	UD	UD	N/A
PN180308.01	No	Yes	0	3	2017	26	1	2017	Winter	No	Yes	5	NT	None	M	UD	N/A	N/A	UD	UD	NT	NT	NT	NT	NT	N/A
PN180308.04	Yes	Yes	N/A	N/A	2016	5	9	2016	Summer	No	Yes	7	NT	Ample	F	**30**	N/A	**35**	UD	UD	UD	UD	UD	UD	UD	N/A
PN180308.21	No	Yes	11	3	2017	11	3	2017	Winter	No	Yes	4	NT	Mild	M	UD	N/A	N/A	UD	UD	NT	NT	NT	NT	NT	N/A
PN180308.10	No	Yes	29	4	2017	29	4	2017	Spring	No	Yes	4	NT	None	M	UD	N/A	N/A	UD	UD	NT	NT	NT	NT	NT	N/A
PN180308.12	No	No	2	4	2017	N/A	N/A	N/A	Spring	Yes	Yes	6	NT	None	F	UD	N/A	N/A	UD	UD	NT	NT	NT	NT	NT	N/A
PN180308.08	No	Yes	N/A	N/A	2016	15	9	2016	Summer	No	Yes	7	NT	None	M	UD	N/A	N/A	UD	UD	NT	NT	NT	NT	NT	N/A
PN180308.15	No	Yes	1	4	2017	30	3	2017	Spring	No	Yes	6	NT	Mild	M	UD	N/A	N/A	UD	UD	NT	NT	NT	NT	NT	N/A
PN180308.06	No	Yes	26	5	2017	26	5	2017	Spring	Yes	Yes	5	NT	Mild	M	UD	N/A	N/A	UD	N/A	NT	NT	NT	NT	NT	N/A
PN180308.09	No	Yes	17	5	2017	17	5	2017	Spring	Yes	Yes	5	NT	None	M	UD	N/A	N/A	UD	UD	NT	NT	NT	NT	NT	N/A
PN180308.18	No	Yes	3	5	2017	3	5	2017	Spring	No	Yes	6	NT	Moderate	M	UD	N/A	N/A	UD	UD	NT	NT	NT	NT	NT	N/A
PN180308.14	No	Yes	14	8	2017	11	8	2017	Summer	No	Yes	6	NT	Mild	M	UD	N/A	N/A	UD	UD	NT	NT	NT	NT	NT	N/A
PN180308.02	No	Yes	17	9	2017	17	9	2017	Summer	No	Yes	6	NT	None	M	UD	N/A	N/A	UD	N/A	NT	NT	NT	NT	NT	N/A
PN180308.11	No	Yes	6	9	2017	5	9	2017	Summer	Yes	Yes	7	NT	Unclear	M	UD	N/A	N/A	UD	UD	NT	NT	NT	NT	NT	N/A
PN180308.20	No	Yes	11	9	2017	11	9	2017	Summer	No	Yes	6	NT	None	M	UD	N/A	N/A	UD	UD	NT	NT	NT	NT	NT	N/A
PN190312.01	No	Yes	18	9	2017	16	9	2017	Summer	No	Yes	6	NT	Mild	M	N/A	UD	N/A	UD	UD	NT	NT	NT	NT	NT	N/A
PN180308.05	No	Yes	5	10	2017	3	10	2017	Autumn	No	Yes	6	NT	Mild	M	UD	N/A	N/A	UD	UD	NT	NT	NT	NT	NT	N/A
PN180308.19	No	Yes	N/A	N/A	2016	19	7	2016	Summer	No	Yes	7	NT	None	M	UD	N/A	N/A	UD	UD	NT	NT	NT	NT	NT	N/A
PN180308.07	No	No	15	10	2017	N/A	N/A	N/A	Autumn	No	Yes	9	NT	Mild	M	UD	N/A	N/A	UD	UD	NT	NT	NT	NT	NT	N/A
PN180308.16	No	Yes	3	10	2017	2	10	2017	Autumn	Yes	Yes	7	NT	Ample	F	UD	N/A	N/A	UD	UD	NT	NT	NT	NT	NT	N/A
PN180308.22	Yes	Yes	21	10	2017	4	10	2017	Spring	No	Yes	5	NT	None	M	**20**	N/A	N/A	**32**	UD	UD	UD	UD	**35**	UD	N/A
PN190326.23	Yes	Yes	12	2	2018	12	2	2018	Winter	No	Yes	5	32.6	None	M	UD	UD	**31**	**33**	UD	UD	UD	UD	UD	UD	N/A
PN190326.24	No	Yes	3	10	2017	2	10	2017	Autumn	N/A	Yes	6	31.6	None	M	N/A	UD	N/A	UD	UD	NT	NT	NT	NT	NT	N/A
PN191213.04	No	Yes	5	2	2018	24	1	2018	Winter	No	Yes	NT	NT	NT	F	N/A	UD	N/A	UD	UD	NT	NT	NT	NT	NT	UD
PN190326.12	No	Yes	22	3	2018	21	3	2018	Spring	N/A	Yes	5	32.6	None	M	N/A	UD	N/A	UD	UD	NT	NT	NT	NT	NT	UD
PN190326.33	No	Yes	9	3	2018	18	2	2018	Winter	No	Yes	6	35.4	None	F	N/A	UD	N/A	UD	UD	NT	NT	NT	NT	NT	N/A
PN190326.36	No	Yes	6	4	2018	6	4	2018	Spring	No	Yes	6	34.1	None	M	N/A	UD	N/A	UD	UD	NT	NT	NT	NT	NT	N/A
PN190326.37	No	Yes	9	4	2018	9	4	2018	Spring	No	Yes	4	32	Mild	M	UD	UD	UD	UD	UD	UD	UD	UD	UD	UD	UD
PN190326.42	No	Yes	6	4	2018	6	4	2018	Spring	No	Yes	7	34	Mild	M	N/A	UD	N/A	UD	UD	NT	NT	NT	NT	NT	N/A
PN190326.43	No	Yes	6	4	2018	6	4	2018	Spring	Yes	Yes	7	34.2	Ample	F	N/A	UD	N/A	UD	N/A	NT	NT	NT	NT	NT	N/A
PN190326.47	No	Yes	13	4	2018	13	4	2018	Spring	Yes	Yes	6	33.2	Mild	M	N/A	UD	N/A	UD	UD	NT	NT	NT	NT	NT	UD
PN191213.01	Yes	Yes	22	4	2018	12	3	2018	Winter	No	Yes	NT	NT	Mild	M	**36**	**32**	UD	UD	UD	UD	UD	UD	UD	UD	UD
PN190326.02	No	Yes	31	5	2018	30	5	2018	Spring	No	Yes	7	34.3	Mild	M	N/A	UD	N/A	UD	UD	NT	NT	NT	NT	NT	N/A
PN190326.04	No	Yes	24	5	2018	24	5	2018	Spring	N/A	Yes	6	NT	Mild	M	UD	N/A	N/A	UD	UD	NT	NT	NT	NT	NT	N/A
PN190326.10	Yes	Yes	12	5	2018	7	5	2018	Spring	N/A	Yes	5	34.7	Ample	M	**25**	**21**	**17**	**23**	**31**	**33**	UD	**34**	**35**	UD	**32**
PN190326.16	No	Yes	8	5	2018	8	5	2018	Spring	No	Yes	4	34.9	Moderate	M	UD	N/A	N/A	UD	**36**	NT	NT	NT	NT	NT	UD
PN190326.45	Yes	Yes	6	5	2018	6	5	2018	Spring	Yes	Yes	6	33.5	Mild	M	UD	**36**	UD	UD	UD	UD	UD	UD	UD	UD	UD
PN190326.08	No	Yes	27	7	2018	27	7	2018	Summer	No	Yes	7	33.1	Mild	M	UD	N/A	N/A	UD	UD	NT	NT	NT	NT	NT	N/A
PN190326.09	No	Yes	2	7	2018	2	7	2018	Summer	N/A	Yes	5	33.6	Mild	M	N/A	UD	N/A	UD	UD	NT	NT	NT	NT	NT	UD
PN190326.34	No	Yes	13	7	2018	12	7	2018	Summer	Yes	Yes	6	32.9	None	M	N/A	UD	N/A	UD	UD	NT	NT	NT	NT	NT	N/A
PN190326.18	No	Yes	8	12	2017	16	10	2017	Autumn	N/A	Yes	7	33.8	Ample	F	N/A	UD	N/A	UD	UD	NT	NT	NT	NT	NT	N/A
PN190326.05	N/A	Yes	13	8	2018	13	8	2018	Summer	No	Yes	7	32.5	None	M	NT	NT	NT	UD	UD	NT	NT	NT	NT	NT	N/A
PN190326.17	No	Yes	3	8	2018	2	8	2018	Summer	No	Yes	6	31.4	Ample	M	N/A	UD	N/A	UD	UD	NT	NT	NT	NT	NT	UD
PN190326.21	No	Yes	N/A	N/A	N/A	4	10	2017	Spring	No	Yes	8	32.8	Ample	F	UD	N/A	N/A	UD	UD	NT	NT	NT	NT	NT	N/A
PN190326.20	No	Yes	1	8	2018	1	8	2018	Summer	No	Yes	8	33.3	Ample	M	UD	N/A	N/A	UD	UD	NT	NT	NT	NT	NT	UD
PN190326.35	No	Yes	18	8	2018	18	8	2018	Summer	No	Yes	6	34	None	M	UD	UD	N/A	UD	UD	NT	NT	NT	NT	NT	UD

PN190326.29	Yes	Yes	12	12	2017	12	12	2017	Autumn	N/A	Yes	8	34.7	Ample	F	**32**	**30**	N/A	**32**	UD	UD	UD	**36**	UD	UD	N/A
PN190326.25	No	Yes	N/A	N/A	N/A	21	11	2017	Autumn	No	Yes	8	34.7	Ample	F	UD	UD	N/A	UD	N/A	NT	NT	NT	NT	NT	N/A
PN190326.26	No	Yes	N/A	N/A	N/A	25	8	2017	Summer	No	Yes	6	34.1	Moderate	M	UD	UD	N/A	UD	UD	NT	NT	NT	NT	NT	UD
PN190326.38	No	Yes	2	8	2018	29	7	2018	Summer	No	Yes	6	32.5	None	M	N/A	UD	N/A	UD	N/A	NT	NT	NT	NT	NT	N/A
PN180308.13	No	Yes	N/A	N/A	2017	30	5	2017	Spring	Yes	Yes	7	NT	None	M	UD	N/A	N/A	UD	UD	NT	NT	NT	NT	NT	N/A
PN190326.41	No	Yes	17	8	2018	16	8	2018	Summer	No	Yes	7	33.9	None	M	UD	UD	N/A	UD	UD	NT	NT	NT	NT	NT	N/A
PN190326.31	No	Yes	N/A	N/A	N/A	5	10	2017	Autumn	Yes	Yes	6	32.3	Mild	F	UD	UD	N/A	UD	UD	NT	NT	NT	NT	NT	UD
PN190326.03	Yes	Yes	4	9	2018	3	9	2018	Summer	N/A	Yes	6	33.5	Moderate	M	**26**	**27**	**22**	**26**	UD	UD	**33**	UD	UD	UD	**36**
PN190326.07	No	Yes	16	9	2018	16	9	2018	Summer	No	Yes	6	35	Mild	M	N/A	UD	N/A	UD	N/A	NT	NT	NT	NT	NT	N/A
PN190326.11	No	Yes	14	9	2018	14	9	2018	Summer	No	Yes	5	33.1	Moderate	M	N/A	UD	N/A	UD	UD	NT	NT	NT	NT	NT	UD
PN190326.13	Yes	Yes	30	9	2018	29	9	2018	Autumn	No	Yes	6	34.5	None	M	**27**	**25**	N/A	**25**	UD	UD	UD	UD	**32**	UD	N/A
PN190326.14	Yes	Yes	16	9	2018	16	9	2018	Summer	No	Yes	7	32.7	None	M	UD	UD	N/A	**23**	UD	UD	**35**	UD	**34**	UD	N/A
PN190326.19	No	Yes	11	9	2018	11	9	2018	Summer	No	Yes	6	35	None	F	N/A	UD	N/A	UD	UD	NT	NT	NT	NT	NT	UD
PN190326.22	No	Yes	16	9	2018	16	9	2018	Summer	No	Yes	6	33.1	Mild	M	N/A	UD	N/A	UD	UD	NT	NT	NT	NT	NT	UD
PN190326.44	Yes	Yes	23	1	2019	23	1	2019	Winter	No	Yes	5	33.7	None	M	**28**	**29**	**27**	**29**	**34**	**30**	UD	UD	UD	UD	N/A
PN190326.27	No	Yes	3	9	2018	3	9	2018	Summer	Yes	Yes	6	33.5	Mild	F	N/A	UD	N/A	UD	UD	NT	NT	NT	NT	NT	UD
PN190326.30	Yes	Yes	16	9	2018	14	9	2018	Summer	Yes	Yes	7	33.5	None	M	UD	**30**	UD	UD	UD	UD	UD	UD	UD	UD	N/A
PN190326.40	No	Yes	20	9	2018	15	9	2018	Summer	No	Yes	5	32.4	None	M	N/A	UD	N/A	UD	UD	NT	NT	NT	NT	NT	UD
PN191213.02	No	Yes	30	9	2018	29	9	2018	Autumn	No	Yes	NT	NT	NT	M	N/A	UD	N/A	UD	UD	NT	NT	NT	NT	NT	UD
PN190326.39	No	Yes	13	2	2019	13	2	2019	Winter	No	Yes	5	33.7	Mild	M	UD	UD	N/A	UD	UD	NT	NT	NT	NT	NT	UD
PN190326.01	Yes	Yes	15	10	2018	15	10	2018	Autumn	No	Yes	6	32.51	Mild	M	UD	**33**	UD	UD	UD	UD	UD	UD	UD	UD	UD
PN190326.06	Yes	Yes	11	10	2018	11	10	2018	Autumn	No	Yes	7	33.8	Moderate	M	UD	**30**	UD	**29**	UD	UD	UD	UD	UD	UD	UD
PN190326.15	No	Yes	2	10	2018	2	10	2018	Autumn	No	Yes	6	32.9	Mild	M	N/A	UD	N/A	UD	UD	NT	NT	NT	NT	NT	N/A
PN190326.32	No	Yes	9	10	2018	9	10	2018	Autumn	No	Yes	7	34.4	Moderate	F	UD	UD	N/A	UD	UD	NT	NT	NT	NT	NT	UD
PN190402	Yes	Yes	2	4	2019	2	4	2019	Spring	Yes	No	7	34.75	Moderate	F	N/A	N/A	**18**	**27**	UD	UD	UD	UD	UD	UD	UD
PN190409	N/A	Yes	9	4	2019	8	4	2019	Spring	Yes	No	5	34.32	Moderate	M	NT	NT	NT	UD	UD	NT	NT	NT	NT	NT	UD
PN190417	N/A	Yes	17	4	2019	17	4	2019	Spring	Yes	No	6	32.67	Mild	M	NT	NT	NT	UD	N/A	NT	NT	NT	NT	NT	N/A
PN191008	N/A	Yes	7	10	2019	6	10	2019	Autumn	No	No	6	33.9	Ample	F	NT	NT	NT	UD	UD	NT	NT	NT	NT	NT	UD
PN191213.03	No	Yes	9	10	2018	9	10	2018	Autumn	No	Yes	NT	NT	NT	F	N/A	UD	N/A	UD	UD	NT	NT	NT	NT	NT	UD
PN181129	No	Yes	29	11	2018	28	9	2018	Autumn	Yes	No	6	NT	Mild	M	UD	N/A	N/A	UD	UD	NT	NT	NT	NT	NT	N/A
PN190326.48	Yes	No	N/A	N/A	2018	N/A	N/A	N/A	Autumn	N/A	Yes	6	32.4	Mild	M	UD	**30**	UD	UD	UD	UD	UD	UD	UD	UD	UD
PN190326.49	Yes	No	N/A	N/A	2018	N/A	N/A	N/A	Autumn	N/A	Yes	7	33.3	Ample	F	UD	**31**	UD	UD	UD	UD	UD	UD	UD	UD	N/A
PN200303	No	Yes	3	3	2020	2	3	2020	Winter	No	No	6	35.4	Moderate	M	UD	N/A	N/A	UD	N/A	NT	NT	NT	NT	NT	N/A
PN200309	Yes	Yes	9	3	2020	8	3	2020	Winter	No	No	6	33.8	None	M	**30**	N/A	**24**	**32**	UD	**33**	UD	UD	UD	UD	UD
PN210408	Yes	Yes	8	4	2021	6	4	2021	Spring	No	No	5	36.2	NT	F	**23**	**18**	**19**	**26**	UD	**24**	**23**	**28**	UD	UD	UD
PN210423	Yes	Yes	23	4	2021	23	4	2021	Spring	Yes	No	6	33.8	NT	M	**25**	**23**	**26**	**26**	UD	UD	**37**	UD	UD	**31**	**32**
PN210910	Yes	Yes	10	9	2021	9	9	2021	Summer	No	No	6	34.3	Moderate	M	**23**	UD	**32**	**23**	UD	UD	UD	UD	UD	UD	UD
PN210920	Yes	Yes	19	9	2021	18	9	2021	Summer	Yes	No	5	31.2	None	M	**37**	**29**	UD	UD	UD	UD	UD	UD	UD	UD	UD
PN210928.01	Yes	Yes	26	9	2021	25	9	2021	Autumn	Yes	No	7	35	Ample	M	**26**	UD	UD	UD	UD	UD	UD	UD	UD	UD	UD
PN210928.02	No	Yes	27	9	2021	26	9	2021	Autumn	No	No	6	34.1	None	M	UD	UD	N/A	UD	N/A	NT	NT	NT	NT	UD	N/A
PN211001	Yes	Yes	30	9	2021	30	9	2021	Autumn	Yes	No	5	44.7	None	M	UD	N/A	**28**	**33**	UD	UD	UD	UD	UD	UD	UD

S2: List of novel sequence names as shown in Figure 1 with accession number and Erasmus bat ID number (Appendix A1) and Rdrp fragment lengths after trimming.

Sequence name	Erasmus ID #	Accession no.	Sequence length (nt)
BatCoV_P.nat_NL_2017-02	PN180308.17	OQ348392	395
BatCoV_P.nat_NL_2017-19	PN180308.22	OQ348393	395
BatCoV_P.nat_NL_2018-26	PN190326.03	OQ348394	395
BatCoV_P.nat_NL_2018-39	PN190326.06	OQ348395	395
BatCoV_P.nat_NL_2018-13	PN190326.10	OQ348396	395
BatCoV_P.nat_NL_2018-34	PN190326.13	OQ348397	395
BatCoV_P.nat_NL_2018-30	PN190326.14	OQ348398	395
BatCoV_P.nat_NL_2018-02	PN190326.23	OQ348399	395
BatCoV_P.nat_NL_2019-01	PN190326.44	OQ348400	395
BatCoV_P.nat_NL_2019-03	PN190402	OQ348401	395
BatCoV_P.nat_NL_2020-02	PN200309	OQ348402	395
BatCoV_P.nat_NL_2021-01	PN210408	OQ348403	395
BatCoV_P.nat_NL_2021-02	PN210423	OQ348404	395
BatCoV_P.nat_NL_2021-03	PN210910	OQ348405	360
BatCoV_P.nat_NL_2021-05	PN210928.01	OQ348406	383

S3: List of reference sequences obtained from GenBank, short names as shown in Figure 1 and *Rdrp* fragment lengths used for alignment.

Full sequence name	Short name	Sequence length (nt)
ON325306.1_MERSr_coronavirus_isolate_Bat-CoV/P.nathusii/Russia/MOW15-22/2015	BatCoV_P.nat_RUS_2015_MOW15-22	395
KC243390.1_Bat_coronavirus_BtCoV/8-724/Pip_pyg/ROU/2009	BatCoV_P.pyg_ROU_2009_8-724	395
GQ259977.1_Coronavirus_P.pipi/VM314/2008/NLD	BatCoV_P.pip_NL_2008_VM314	332
KC243391.1_Bat_coronavirus_BtCoV/8-691/Pip_nat/ROU/2009	BatCoV_P.nat_ROU_2009_8-691	395
KC243392.1_Bat_coronavirus_BtCoV/UKR-G17/Pip_nat/UKR/2011	BatCoV_P.nat_UKR_2011_UKR-G17	395
NC_019843.3_MERSr_coronavirus_isolate_HCoV-EMC/2012	HCoV-MERS-CoV_EMC_2012	395
KC545383.1_ErinaceusCoV/2012-174/GER/2012	EriCoV_GER_2012-174	395
KJ473821.1_BtVs-BetaCoV/SC2013	BatCoV_V.sup_SC2013	395
KX442565.1_Hypsugo_bat_coronavirus_HKU25_isolate_NL140462	BatCoV_H.pul_HKU25	395
NC_034440.1_Bat_coronavirus_isolate_PREDICT/PDF-2180	BatCoV_PREDICT_PDF-2180	395
KC869678.4_Coronavirus_Neoromicia/PML-PHE1/RSA/2011	BatCoV_N.cap_RSA_2011_PML-PHE1	395
MG596802.1_MERSr-CoV_isolate_Bat-CoV/H.savii/Italy/206645-40/2011	BatCoV_H.sav_IT_2011_206645-40	395
MG596803.1_MERSr-CoV_isolate_Bat-CoV/P.khulii/Italy/206645-63/2011	BatCoV_P.khu_IT_2011_206645-63	395
NC_009019.1_Tylonycteris_bat_coronavirus_HKU4	BatCoV_T.pac_HKU4	395
DQ249217.1_Bat_coronavirus_HKU5_isolate_HKU5-1	BatCoV_P.pip_HKU5-1	395
NC_009021.1_Rousettus_bat_coronavirus_HKU9	BatCoV_R.lec_HKU9	395
KF686346.1_Human_coronavirus_HKU1_strain_HKU1/human/USA/HKU1-12/2010	HCoV-HKU1_USA_2010_HKU1-12	395
FJ647226.1_Murine_coronavirus_MHV-JHM.IA	MurCoV_MHV-JHM.IA	395
KY370043.1_Rodent_coronavirus_isolate_RtRn-CoV/YN2013	RtCoV_YN2013	395
MN306053.1_Human_coronavirus_OC43_strain_HCoV_OC43/Seattle/USA/SC9430/2018	HCoV-OC43_USA_2018_SC9430	395
FJ425189.1_Sambar_deer_coronavirus_US/OH-WD388/1994	SCoV_S.uni_USA_1994_OH-WD388	395
KY370044.1_Rodent_coronavirus_isolate_RtAs-CoV/IM2014	RtCoV_IM2014	395
KY370046.1_Rodent_coronavirus_isolate_RtMruf-CoV-2/JL2014	RtCoV_2_JL2014	395
NC_030886.1_Rousettus_bat_coronavirus_isolate_GCCDC1_356	BatCoV_R.lec_GCCDC1_356	395
AY274119.3_SARS_coronavirus_Tor2	HCoV-SARS-CoV_Tor2	395
KP886808.1_Bat_SARS-like_coronavirus_YNLF_31C	BatCoV_R.fer_YNLF_31C	395
FJ588686.1_Bat_SARS_CoV_Rs672/2006	BatCoV_R.sin_2006_Rs672	395
KY938558.1_Sarbecovirus_sp._strain_16BO133	BatCoV_R.fer_16BO133	395
MN908947.3_Severe_acute_respiratory_syndrome_coronavirus_2_isolate_Wuhan-Hu-1	HCoV-SARS-CoV-2_Wuhan-Hu-1	395
NC_014470.1_Bat_coronavirus_BM48-31/BGR/2008	BatCoV_R.bla_BGR_2008_BM48-31	395
NC_025217.1_Bat_Hp-betacoronavirus/Zhejiang2013	BatCoV_H.pra_Zhejiang_2013	395

S4: Sequence similarity matrix showing percent similarity between all trimmed sequences obtained and reference sequences as shown in figure 1.

	1	2	3	4	5	6	7	8	9	10	11	12	13	14	15	16	17	18	19	20	21	22	23
1. BatCoV_P.nat_NL_2017-02		100	98.7	98.2	99.2	98.2	98.7	98.7	98	99.2	99	98.5	99	89.4	95.7	99	98.5	83.5	98.2	98.2	88.9	78.5	83.3
2. BatCoV_P.nat_NL_2017-19	100		98.7	98.2	99.2	98.2	98.7	98.7	98	99.2	99	98.5	99	89.4	95.7	99	98.5	83.5	98.2	98.2	88.9	78.5	83.3
3. BatCoV_P.nat_NL_2018-26	98.7	98.7		97.5	98	97.5	97.5	97.5	96.7	98	98.2	97.7	97.7	88.6	94.4	98.2	97.7	82.5	97	97	88.1	77.5	82.8
4. BatCoV_P.nat_NL_2018-39	98.2	98.2	97.5		98	97	97	97.5	96.7	98	98.2	97.7	97.2	89.1	94.9	97.7	98.2	83.1	97	97	88.4	77.3	82.6
5. BatCoV_P.nat_NL_2018-13	99.2	99.2	98	98		97.5	98	98.5	97.7	99.5	98.7	98.2	98.2	89.1	95.4	98.2	98.2	83.8	98	98	88.1	78	83.3
6. BatCoV_P.nat_NL_2018-34	98.2	98.2	97.5	97	97.5		97	97	96.7	97.5	97.7	97.7	98.2	88.4	94.4	98.2	98.2	82.5	97	97.5	88.6	79	84.6
7. BatCoV_P.nat_NL_2018-30	98.7	98.7	97.5	97	98	97		98	97.2	98	97.7	97.7	97.7	88.9	94.9	98.2	97.2	82.5	97	97	88.4	79.5	83
8. BatCoV_P.nat_NL_2018-02	99	99	97.7	97.7	98.7	97.2	98.2		98.7	99	98.2	98.2	97.7	89.6	95.9	98.7	97.7	82.8	97.5	97.5	88.6	77.7	84.1
9. BatCoV_P.nat_NL_2019-01	98.2	98.2	97	97	98	97	97.5	99.2		98.2	97.5	97.5	97	89.1	95.2	98	97	82.3	96.7	96.7	88.9	78	84.3
10. BatCoV_P.nat_NL_2019-03	99.2	99.2	98	98	99.5	97.5	98	99.2	98.5		98.7	98.2	98.2	89.1	95.9	98.7	98.2	83.8	98	98	88.1	77.7	83.5
11. BatCoV_P.nat_NL_2020-02	99	99	98.2	98.2	98.7	97.7	97.7	98.5	97.7	98.7		98.5	98	89.4	95.2	98.5	98.5	83.3	97.7	97.7	88.4	78	83.8
12. BatCoV_P.nat_NL_2021-01	98.5	98.5	97.7	97.7	98.2	97.7	97.7	98.5	97.7	98.2	98.5		98.5	89.4	95.2	98.5	98.5	82.8	97.7	97.7	88.9	78.5	84.3
13. BatCoV_P.nat_NL_2021-02	99	99	97.7	97.2	98.2	98.2	97.7	98	97.2	98.2	98	98.5		88.4	94.7	98.5	98.5	82.5	97.7	97.7	88.6	78.7	83.3
14. BatCoV_P.nat_NL_2021-03	89.4	89.4	88.6	89.1	89.1	88.4	88.9	89.9	89.4	89.1	89.4	89.4	88.4		91.9	89.4	89.1	89	88.4	88.1	81	71.6	76.5
15. BatCoV_P.nat_NL_2021-05	95.7	95.7	94.4	94.9	95.4	94.4	94.9	96.2	95.4	95.9	95.2	95.2	94.7	91.9		95.7	94.9	85.4	94.4	94.4	85.6	75.7	81
16. BatCoV_P.nat_RUS_MOW15-22/2015	99	99	98.2	97.7	98.2	98.2	98.2	99	98.2	98.7	98.5	98.5	98.5	89.4	95.7		98.5	82.8	97.2	97.2	89.4	78.7	84.3
17. BatCoV_P.pyg_ROU_2009_8-724	98.5	98.5	97.7	98.2	98.2	98.2	97.2	98	97.2	98.2	98.5	98.5	98.5	89.1	94.9	98.5		83	97.7	97.7	88.6	78.7	84.1
18. BatCoV_P.pip_NL_2008_VM314	83.5	83.5	82.5	83.1	83.8	82.5	82.5	83	82.5	83.8	83.3	82.8	82.5	90	85.4	82.8	83		82.5	82.8	73.7	65.3	70.6
19. BatCoV_P.nat_ROU_2009_8-691	98.2	98.2	97	97	98	97	97	97.7	97	98	97.7	97.7	97.7	88.4	94.4	97.2	97.7	82.5		99.5	88.4	78.5	84.3
20. BatCoV_P.nat_UKR_2011_UKR-G17	98.2	98.2	97	97	98	97.5	97	97.7	97	98	97.7	97.7	97.7	88.1	94.4	97.2	97.7	82.8	99.5		88.1	78.7	84.1
21. HCoV_MERS-CoV_EMC/2012	88.9	88.9	88.1	88.4	88.1	88.6	88.4	88.9	89.1	88.1	88.4	88.9	88.6	81	85.6	89.4	88.6	73.7	88.4	88.1		81.5	84.1
22. EriCoV_GER_2012-174	78.5	78.5	77.5	77.3	78	79	79.5	78	78.2	77.7	78	78.5	78.7	71.6	75.7	78.7	78.7	65.3	78.5	78.7	81.5		77
23. BatCoV_V.sup_SC2013	83.3	83.3	82.8	82.6	83.3	84.6	83	84.3	84.6	83.5	83.8	84.3	83.3	76.5	81	84.3	84.1	70.6	84.3	84.1	84.1	77	
24. BatCoV_H.pul_HKU25	81	81	80.3	79.8	80.8	81.8	81.3	80.5	80.8	80.8	80	81.3	81.8	72.9	77.5	81	80.8	67.1	81.5	81.8	84.1	79	82.3
25. BatCoV_PREDICT/PDF-2180	86.8	86.8	86.1	85.9	86.1	87.6	87.1	86.8	87.1	86.1	86.6	87.6	87.3	79.7	83.8	87.3	87.3	73.4	86.8	87.1	90.1	82.5	82.8
26. BatCoV_N.cap_RSA_2011_PML-PHE1	87.1	87.1	86.3	86.1	86.3	86.8	86.8	87.1	86.8	86.3	86.6	86.6	86.8	79.2	84.6	87.1	86.3	72.7	86.6	86.8	91.9	81.5	83.8
27. BatCoV_H.sav_IT_2011_206645-40	84.6	84.6	84.1	83.3	83.8	85.8	84.1	84.6	84.6	83.8	85.1	85.6	85.1	77	81.5	85.1	84.6	70.1	84.6	84.8	85.8	79.5	84.1
28. BatCoV_P.khu_IT_2011_206645-63	84.8	84.8	84.3	83.6	84.1	85.6	84.3	84.8	84.8	84.1	84.8	85.8	85.6	77.2	81.3	85.3	84.8	70.4	84.8	85.1	86.8	80	83.3
29. BatCoV_T.pac_HKU4	77	77	76.7	75.8	76.5	77.2	77.5	77.2	78	76.5	77.2	78	77.7	70.4	73.4	77.7	77.2	63.3	76.5	76.2	78	79.2	78.7
30. BatCoV_P.pip_HKU5-1	81	81	80.5	80.1	80.3	82	80.5	81.3	82	80.5	80.8	81.3	81.8	73.2	78.5	81.5	81.5	66.8	80.8	80.8	83.3	80	80
31. BatCoV_R.lec_HKU9	65.3	65.3	64.8	64.1	64.6	64.8	66.1	65.8	66.1	65.1	64.8	65.1	64.6	61	63.3	65.8	64.6	56.5	64.3	64.8	66.1	63.5	63
32. HCoV_HKU1_USA_2010_HKU1-12	66.1	66.1	65.6	65.2	65.8	66.1	67.6	67.1	67.6	66.3	66.1	66.6	66.1	61.3	63.5	66.8	66.3	56.5	65.8	66.3	65.6	67.6	66.3
33. MurCoV_MHV-JHM.IA	65.6	65.6	64.6	64.6	65.1	64.6	66.3	65.6	66.1	64.8	65.1	65.6	65.6	60.8	63.8	65.8	65.6	55.7	64.1	64.6	63	66.6	63.5
34. RtCoV_YN2013	65.1	65.1	64.1	63.6	64.6	64.6	65.6	65.1	65.1	64.3	64.6	64.8	65.1	60.5	61.5	65.3	64.8	54.9	64.8	65.1	64.8	66.8	66.3
35. HCoV_OC43_USA_2018_SC9430	65.3	65.3	64.6	63.9	64.6	66.3	66.8	65.8	66.3	65.1	64.8	65.1	65.3	60.5	64.1	66.1	65.1	54.7	65.1	65.6	65.1	68.6	64.8
36. SCoV_S.uni_USA_1994_OH-WD388	65.3	65.3	64.6	63.9	64.6	66.1	66.8	65.8	66.1	65.1	64.8	65.1	65.3	60.5	64.1	66.1	65.1	54.9	64.8	65.3	64.3	68.6	65.1
37. RtCoV_IM2014	65.1	65.1	64.3	63.6	64.6	65.8	65.6	64.1	64.3	63.8	64.6	64.8	65.1	59.5	62.8	64.8	64.8	55.9	64.6	65.1	62.8	66.8	63
38. RtCoV_2/JL2014	65.6	65.6	64.6	64.6	65.1	63.8	65.6	66.1	66.1	65.3	65.3	65.1	64.8	60.8	63.3	65.8	65.3	55.9	64.6	64.3	64.6	66.6	67.1
39. BatCoV_R.lec_GCCDC1_356	61.5	61.5	60.8	61.1	61	61.3	62	62.3	62.3	61.5	61.3	62.3	61.8	57.2	60	62	61.5	54.4	61.5	61.5	61.8	62.8	59.2
40. HCoV_SARS_CoV_Tor2	61.3	61.3	60.8	61.9	62.5	61.8	60.8	63.8	64.6	63.5	62.5	62.8	61.3	57.7	61.3	62.3	62.3	52.9	63	63.3	62.5	62.8	64.6
41. BatCoV_R.fer_YNLF_31C	61.5	61.5	61	62.1	62.8	62	61	64.1	64.8	63.8	62.8	63	61.5	58	61.5	62.5	62.5	53.2	63.3	63.5	62.8	63	64.8
42. BatCoV_R.sin_2006_Rs672	63.5	63.5	63	63.9	64.8	64.1	62.5	66.1	66.3	65.8	64.8	65.1	63.5	60	63.5	64.6	64.6	57.2	65.3	65.6	64.1	64.3	65.3
43. BatCoV_R.fer_16BO133	64.8	64.8	64.3	65.2	66.1	65.8	63.8	67.3	67.6	67.1	65.8	65.8	64.8	60.3	64.8	66.3	66.3	56.2	66.6	66.8	66.3	66.6	67.3
44. HCoV_SARS_CoV_2_Wuhan-Hu-1	64.3	64.3	63.5	62.4	62.5	65.3	62.5	63.8	64.6	63.5	62	62.3	64.3	56.7	61	65.8	62.5	54.9	62.8	63.3	64.6	65.3	63.3
45. BatCoV_R.bla_BGR_2008_BM48-31	65.6	65.6	65.1	63.4	64.1	66.6	64.6	65.3	66.1	65.1	63.5	64.6	66.3	59.5	62.3	67.1	64.1	56.5	64.3	64.3	66.8	68.1	63.8
46. BatCoV_H.pra_Zhejiang_2013	66.6	66.6	66.1	66.2	66.1	66.3	66.3	66.3	66.1	66.1	66.8	66.1	66.3	59.5	63.8	66.1	65.8	55.4	66.1	66.1	67.1	68.9	66.6

	24	25	26	27	28	29	30	31	32	33	34	35	36	37	38	39	40	41	42	43	44	45
1. BatCoV_P.nat_NL_2017-02	81	86.8	87.1	84.6	84.8	77	81	65.3	64.8	64.3	64.7	64.3	64.2	64.7	65.2	61.5	61	61.2	62.9	64.2	63.8	65.1
2. BatCoV_P.nat_NL_2017-19	81	86.8	87.1	84.6	84.8	77	81	65.3	64.8	64.3	64.7	64.3	64.2	64.7	65.2	61.5	61	61.2	62.9	64.2	63.8	65.1
3. BatCoV_P.nat_NL_2018-26	80.3	86.1	86.3	84.1	84.3	76.7	80.5	64.8	64.3	63.3	63.7	63.6	63.4	64	64.2	60.8	60.5	60.7	62.4	63.7	63.1	64.6
4. BatCoV_P.nat_NL_2018-39	79.8	85.9	86.1	83.3	83.6	75.8	80.1	64.1	63.9	63.4	63.3	62.9	62.8	63.3	64.3	61.1	61.4	61.7	63.3	64.5	62.2	63.2
5. BatCoV_P.nat_NL_2018-13	80.8	86.1	86.3	83.8	84.1	76.5	80.3	64.6	64.5	63.8	64.2	63.6	63.4	64.2	64.7	61	62.1	62.3	64	65.3	62.5	64.1
6. BatCoV_P.nat_NL_2018-34	81.8	87.6	86.8	85.8	85.6	77.2	82	64.8	64.8	64.4	64.4	65.3	65.1	65.5	63.8	61.3	61.5	61.7	63.4	65.2	64.8	66.1
7. BatCoV_P.nat_NL_2018-30	81.3	87.1	86.8	84.1	84.3	77.5	80.5	66.1	66.1	64.9	65.2	65.7	65.5	65.2	65.2	62	60.8	61	62.2	63.5	62.5	64.6
8. BatCoV_P.nat_NL_2018-02	80.3	86.6	86.8	84.3	84.6	77	81	65.6	65.8	64.3	64.7	64.6	64.4	63.6	65.5	62	62.6	62.8	64.5	66.1	63.1	64.9
9. BatCoV_P.nat_NL_2019-01	80.5	86.8	86.6	84.3	84.6	77.7	81.8	65.8	66	64.5	64.5	64.9	64.7	63.9	65.7	62	63.3	63.6	64.8	66.3	63.8	65.7
10. BatCoV_P.nat_NL_2019-03	80.8	86.1	86.3	83.8	84.1	76.5	80.5	65.1	65	63.5	64	64.1	63.9	63.6	65	61.5	62.6	62.8	64.5	66.1	63.1	64.9
11. BatCoV_P.nat_NL_2020-02	80	86.6	86.6	85.1	84.8	77.2	80.8	64.8	64.8	63.8	64.2	63.8	63.7	64.2	65	61.3	62.1	62.3	64	65	62	63.5
12. BatCoV_P.nat_NL_2021-01	81.3	87.6	86.6	85.6	85.8	78	81.3	65.1	65.3	64.3	64.5	64.1	63.9	64.5	64.7	62.3	62.3	62.6	64.3	65	62.3	64.6
13. BatCoV_P.nat_NL_2021-02	81.8	87.3	86.8	85.1	85.6	77.7	81.8	64.6	64.8	64.3	64.7	64.3	64.2	64.7	64.5	61.8	61	61.2	62.9	64.2	63.8	65.8
14. BatCoV_P.nat_NL_2021-03	72.9	79.7	79.2	77	77.2	70.4	73.2	60.7	60	59.6	60.2	59.6	59.5	59.2	60.5	56.9	57.3	57.5	59.3	59.5	56.7	59.5
15. BatCoV_P.nat_NL_2021-05	77.5	83.8	84.6	81.5	81.3	73.4	78.5	63.3	62.3	62.5	61.2	63.1	62.9	62.6	63	60	60.3	60.6	62.3	63.8	60.6	62.1
16. BatCoV_P.nat_RUS_MOW15-22/2015	81	87.3	87.1	85.1	85.3	77.7	81.5	65.8	65.5	64.5	65	65.1	64.9	64.6	65.5	62	61.5	61.8	63.4	65.5	64.8	66.4
17. BatCoV_P.pyg_ROU_2009_8-724	80.8	87.3	86.3	84.6	84.8	77.2	81.5	64.6	65	64.3	64.5	64.1	63.9	64.5	65	61.5	61.8	62.1	63.8	65.5	62.5	64.1
18. BatCoV_P.pip_NL_2008_VM314	67.1	73.4	72.7	70.1	70.4	63.3	66.8	56.5	55.3	55.4	54.7	54.7	54.9	55.7	55.7	54.4	52.5	52.8	56.4	55.5	54.9	56.3
19. BatCoV_P.nat_ROU_2009_8-691	81.5	86.8	86.6	84.6	84.8	76.5	80.8	64.3	64.5	63.7	64.5	64.1	64	64.2	64.4	61.5	62.6	62.8	64.5	65.8	62.8	64.3
20. BatCoV_P.nat_UKR_2011_UKR-G17	81.8	87.1	86.8	84.8	85.1	76.2	80.8	64.8	65	64.2	64.7	64.6	64.5	64.7	64.1	61.5	62.8	63.1	64.8	66	63.3	64.3
21. HCoV_MERS-CoV_EMC/2012	84.1	90.1	91.9	85.8	86.8	77.8	83.3	66.1	64.3	62.9	64.3	64.1	63.3	62.6	64.4	61.8	62.2	62.5	63.4	65.7	64.1	66.8
22. EriCoV_GER_2012-174	79	82.5	81.5	79.5	80	79.2	80	63.5	66.4	65.4	66.3	67.6	67.8	66.7	66.1	62.8	62.8	63	64	66.2	65.3	67.8
23. BatCoV_V.sup_SC2013	82.3	82.8	83.8	84.1	83.3	78.7	80	63	66.2	63.4	66	64.6	64.9	62.9	66.9	59.2	64.1	64.3	64.8	66.8	63.1	63.8
24. BatCoV_H.pul_HKU25		83.5	83.5	83.3	83.8	76.5	82.3	63.9	64.6	62.6	62	60.5	61.3	60.8	62.8	60	64.1	64.3	65.8	65.9	65.3	68.4
25. BatCoV_PREDICT/PDF-2180	83.5		90.9	86.1	86.3	78.7	84.8	65.6	66.7	64.3	64.5	65.1	64.8	63.1	64.3	63.8	63.5	63.8	64.2	65.7	65.1	67.3
26. BatCoV_N.cap_RSA_2011_PML-PHE1	83.5	90.9		86.6	86.6	77.6	83	65.8	66	63.9	64	66.6	64.8	64.2	66.2	63.4	64.2	64.5	64.2	66	65.8	66.3
27. BatCoV_H.sav_IT_2011_206645-40	83.3	86.1	86.6		99	79	82	65.3	64.6	64.4	64.6	65.3	64.1	63	63.8	60	62.5	62.3	62.8	62.8	63.7	65.8
28. BatCoV_P.khu_IT_2011_206645-63	83.8	86.3	86.6	99		78.7	81.5	65.3	64.8	64.6	65.2	65.6	63.8	63.3	64.3	60.5	62.5	62.3	62.8	62.5	63.7	66.8
29. BatCoV_T.pac_HKU4	76.5	78.7	78	79	78.7		80.8	64.5	68.2	64.7	66.6	65.7	66	66.2	68.7	65.2	64.3	64.6	64.3	66.1	65.2	67.6
30. BatCoV_P.pip_HKU5-1	82.3	84.8	83	82	81.5	80.8		63.7	64.7	64.5	62.9	64.1	63.6	62.7	63.3	63.5	64.7	65	64.3	64.8	63.5	67.3
31. BatCoV_R.lec_HKU9	64.1	65.6	65.8	65.3	65.3	64.8	64.1		69.6	64.3	68.4	67.7	67.4	65.3	67.3	77.2	64.3	64.3	64.3	64.8	67.1	67.6
32. HCoV_HKU1_USA_2010_HKU1-12	64.6	68.1	66.3	64.6	64.8	68.9	65.8	70.1		83.3	85.1	82.3	82.3	79.7	82.5	66.1	62.7	62.4	62.2	62.4	65.2	64.9
33. MurCoV_MHV-JHM.IA	62.8	65.6	64.1	64.6	64.8	65.1	64.8	64.3	83.3		87.3	83.5	82.8	85.1	83.3	66.6	61.8	61.5	61	60	64.7	62.5
34. RtCoV_YN2013	62.3	64.8	64.3	64.8	65.3	67.1	63.5	68.4	85.1	87.3		85.1	85.1	84.6	84.8	67.1	62.9	62.6	62.4	62.1	64.7	64.4
35. HCoV_OC43_USA_2018_SC9430	60.5	66.1	67.6	66.3	66.6	66.1	64.6	68.4	82.3	83.5	85.1		97	85.8	84.1	66.4	60.8	60.5	60	61.5	62.5	62
36. SCoV_S.uni_USA_1994_OH-WD388	61.3	65.8	64.8	64.1	63.8	66.3	64.1	68.1	82.3	82.8	85.1	97		85.6	84.8	65.1	61	60.8	60.3	61.3	64.3	62
37. RtCoV_IM2014	60.8	63.3	64.6	63	63.3	66.3	63	65.3	79.7	85.1	84.6	85.8	85.6		81.8	65.1	63.3	63	62.5	61.5	66.1	64.5
38. RtCoV_2/JL2014	62.8	64.8	66.3	63.8	64.3	69.4	63.8	67.3	82.5	83.3	84.8	84.1	84.8	81.8		63.3	62.5	62.3	62	63.1	65.3	65.6
39. BatCoV_R.lec_GCCDC1_356	60	63.8	63.5	60	60.5	65.3	63.8	77.2	66.1	66.6	67.1	66.6	65.1	65.1	63.3		64.1	64.4	63.9	63.9	65	65.7
40. HCoV_SARS_CoV_Tor2	64.1	63.5	64.6	62.5	62.5	64.8	65.1	64.3	63.3	61.8	63	60.8	61	63.3	62.5	64.3		99.7	97.2	89.1	87.8	87.6
41. BatCoV_R.fer_YNLF_31C	64.3	63.8	64.8	62.3	62.3	65.1	65.3	64.3	63	61.5	62.8	60.5	60.8	63	62.3	64.6	99.7		97.5	89.4	87.8	87.8
42. BatCoV_R.sin_2006_Rs672	66.3	64.6	64.6	62.8	62.8	64.8	64.3	64.3	62.8	61	62.5	60	60.3	62.5	62	64.1	97.2	97.5		89.6	88.4	88.1
43. BatCoV_R.fer_16BO133	66.6	66.1	66.3	62.8	62.5	66.6	64.8	64.8	63	60	62.3	61.8	61.3	61.5	63.3	64.1	89.1	89.4	89.6		87.8	86.1
44. HCoV_SARS_CoV_2_Wuhan-Hu-1	65.3	65.1	66.3	64.1	64.1	65.3	63.5	67.1	65.8	65.1	65.1	62.8	64.8	66.6	65.8	65.3	87.8	87.8	88.4	87.8		88.1
45. BatCoV_R.bla_BGR_2008_BM48-31	68.6	67.3	66.3	66.3	67.3	68.1	67.6	67.6	65.6	62.5	65.1	62.3	62	64.8	66.1	66.1	87.6	87.8	88.1	86.1	88.1	
46. BatCoV_H.pra_Zhejiang_2013	65.1	68.4	67.6	67.1	66.6	67.6	66.1	66.6	68.1	65.3	66.1	64.8	63.3	65.6	68.6	63	68.6	68.4	67.8	69.1	69.1	68.1
